# Impacts of Climate Change on Health and Health Services in Northern New South Wales, Australia: A Rapid Review

**DOI:** 10.3390/ijerph20136285

**Published:** 2023-07-03

**Authors:** Grace W. Lee, Kristina Vine, Amba-Rose Atkinson, Michael Tong, Jo Longman, Alexandra Barratt, Ross Bailie, Sotiris Vardoulakis, Veronica Matthews, Kazi Mizanur Rahman

**Affiliations:** 1 University of Sydney, University Centre for Rural Health, Lismore, NSW 2480, Australia; g.lee@sydney.edu.au (G.W.L.); kristina.vine@sydney.edu.au (K.V.); ambarose.atkinson@uq.net.au (A.-R.A.); jo.longman@sydney.edu.au (J.L.); veronica.matthews@sydney.edu.au (V.M.); 2 School of Public Health, Faculty of Medicine and Health, University of Sydney, Camperdown, NSW 2006, Australia; alexandra.barratt@sydney.edu.au; 3 Healthy Environments And Lives (HEAL) National Research Network, Canberra, ACT 2601, Australia; michael.tong@anu.edu.au (M.T.); ross.bailie@sydney.edu.au (R.B.); sotiris.vardoulakis@anu.edu.au (S.V.); 4 School of Public Health, Faculty of Medicine, the University of Queensland, St. Lucia, QLD 4072, Australia; 5 College of Health and Medicine, The Australian National University, Canberra, ACT 2601, Australia; 6 Sydney Environment Institute, University of Sydney, Camperdown, NSW 2006, Australia; 7 School of Medicine, Faculty of Medicine and Health, University of Sydney, Camperdown, NSW 2006, Australia

**Keywords:** climate change, health impact, health services, Northern New South Wales, Australia

## Abstract

Climate change is exposing populations to increasing temperatures and extreme weather events in many parts of Australia. To prepare for climate challenges, there is a growing need for Local Health Districts (LHDs) to identify potential health impacts in their region and strengthen the capacity of the health system to respond accordingly. This rapid review summarised existing evidence and research gaps on the impact of climate change on health and health services in Northern New South Wales (NSW)—a ‘hotspot’ for climate disaster declarations. We systematically searched online databases and selected 11 peer-reviewed studies published between 2012–2022 for the Northern NSW region. The most explored health outcome was mental health in the aftermath of floods and droughts, followed by increased healthcare utilisation due to respiratory, cardiovascular and mortality outcomes associated with bushfire smoke or heat waves. Future research directions were recommended to understand: the compounding impacts of extreme events on health and the health system, local data needs that can better inform models that predict future health risks and healthcare utilisation for the region, and the needs of vulnerable populations that require a whole-of-system response during the different phases of disasters. In conclusion, the review provided climate change and health research directions the LHD may undertake to inform future adaptation and mitigation policies and strategies relevant to their region.

## 1. Introduction

The Sixth Assessment Report of the IPCC reiterated unequivocally that greenhouse gas emissions from anthropogenic activities warmed the atmosphere, ocean, and land [1]. For Australia, the environmental consequences of climate change are already evident. Since 1910, Australia’s land surface temperature has warmed by 1.4 °C, resulting in higher temperatures and more extreme heat waves [2]. Over the decades, more intense and frequent fire weather has been observed across the country, while the country’s southwest and southeast have experienced a decline in rainfall [2]. Oceans around Australia have risen significantly higher than the global average since 1880 and have warmed by 1 °C [2]. Australia will continue to experience increases in temperatures, heat extremes, dangerous fire weather days and fire seasons [2,3]. Extreme events, such as heat waves, droughts, floods, and dust storms, are projected to become more widespread and more intense nationally [2,3]. Continual warming of the climate will have pervasive and irreversible consequences for the environment and ecosystem, which in turn will have impacts on human health and the systems that support health.

In 2009, the Lancet Commission highlighted climate change as “the biggest global health threat of the 21st century” [4] and extended this in 2015 to add that “tackling climate change could be the greatest global health opportunity” [5]. In Australia, the continued lack of an ambitious national policy to reduce greenhouse gas emissions, coupled with inadequate climate change and health adaptation planning, increasingly puts the health of the country’s population and environment at risk [6,7]. At the regional level, there is a growing recognition that tackling the health impacts of climate change will require local action from health organisations.

In the state of New South Wales (NSW), Local Health Districts (LHDs) are responsible for developing the capacity of the healthcare workforce and providing health services in defined geographical areas [8]. The LHDs will have an important role in protecting, promoting and supporting the health of the communities they serve under a changing climate at the regional level [9].

**Figure 1:**
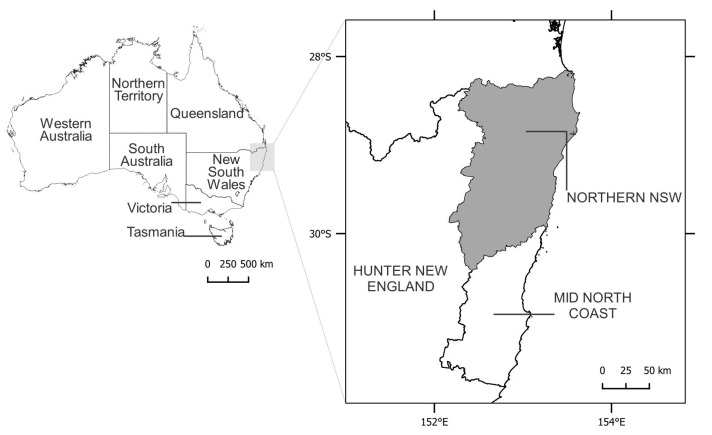
Map showing the study region of Northern NSW Local Health District (shaded in grey).

This paper reports on a rapid review of the Northern New South Wales Local Health District (NNSW LHD) to outline the evidence of climate-related health risks in Northern NSW (Figure 1). Northern NSW is a known ‘hotspot’ for climate disaster declarations for floods and bushfires in Australia. The region has experienced three protracted droughts in recorded history, including the Millennium Drought (1997–2010), which caused serious impacts on agricultural production, biodiversity, bushfire regimes, and water availability [10]. The subsequent decade saw major floods occurring in 2017, followed by two devastating floods within a one-month period in 2022 [11]. In the summer of 2019–2020, the region was also affected by the unprecedented bushfires that burnt 5 million hectares of land on the eastern seaboard of NSW [12].

**Figure 2:**
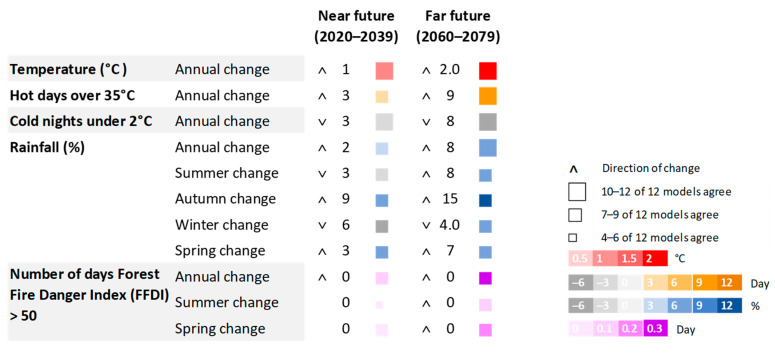
Model agreement and predicted direction and magnitude of change in temperature, hot days, cold nights, rainfall, and fire danger in Northern NSW in the near and far future.

Figure 2 shows the climate projections for Northern NSW in the near (2020–2039) and far (2060–2079) future, modelled by the NSW and ACT Regional Climate Modelling project (NARCliM) [13]. The NARCliM projections were based on the 2010 Coupled Model Intercomparison Project 3 (CMIP3) [13]. The near and far future climate impacts were based on the A2 emissions scenario, which describes a future world of moderate population growth and economic and technological development [14]. Northern NSW will expect a warming trend and a greater number of days over 35 °C. Some hotspots have been identified in inland areas with increases of up to 10 and 20 days above 35 °C in the near and far future, respectively [15]. Rainfall is highly variable spatially and temporally across the region. The annual, spring, and autumn rainfall will increase in the near and far future, whilst winter rainfall will decrease in both future scenarios [15]. Overall, fire weather will intensify in the spring and summer periods. Sea levels on the East Coast of Australia are also projected to rise by 0.13 to 0.15 m by 2030 and 0.55 to 0.63 m by 2090 [16]. Given that the East Coast experiences storm surges and wind waves, climate change will exacerbate these coastal extremes.

The projected changes in climate will have implications for human health. Vulnerability to climate change in Northern NSW derives from many factors: the settlement of major populations alongside rivers or the coasts served by transport infrastructure that are also at risk of inundation [17], an increasing population at risk of exposure to climate-related hazards (i.e., 9% growth to reach 335,872 in 2041 [18]), and a considerable subpopulation that is sensitive to the effects of climate change. Northern NSW is ageing, where the proportion of people over 65 is expected to grow by 7% to make up 31% of the population by 2041 [18]. A high proportion (approximately 40%) of the population is among the most socio-economically disadvantaged in NSW [19]. Northern NSW is also home to a high proportion of marginalised communities, including Aboriginal and Torres Strait Islander people (approximately 4.4% compared to 3.8% of the Australian population overall [20]), who are disproportionately affected by the negative health consequences of climate change [21].

This rapid review summarises the evidence on the impact of climate change on health and health services in Northern NSW. The purpose is to identify evidence gaps and outline future areas of research that the NNSW LHD may undertake to understand climate-related health risks for the region. We expect the review to offer guidance on the LHD’s strategic planning of research for the purpose of improving population health and delivering a climate-resilient health system and workforce.

## 2. Materials and Methods

We followed the Preferred Reporting Items for Systematic Review and Meta-Analysis (PRISMA) guidelines [22] to systematically select and summarise the findings of studies that address clearly formulated research questions. The key steps of the rapid review were: (1) Conduct a systematic search of the literature; (2) Undertake a quality assessment; (3) Categorise and summarise the evidence; and (4) Identify evidence gaps and provide recommendations for future research.

### 2.1. Research Questions

We formulated two research questions to guide the search strategy for the rapid review: (1) What are the observed and predicted health impacts of climate change on health for Northern NSW?; (2) What are the observed and predicted impacts of climate change on health services for Northern NSW?

### 2.2. Search Strategy

We formulated a search strategy based on what is known about the relationship between climate change and health. Many reviews have already documented the direct and indirect health impact of climate change globally and in Australia [5,6,23,24,25,26]: from threats of injury and death during flooding and bushfires to increased respiratory and cardiovascular morbidities and mortalities caused by heat waves, bushfires, dust storms, or extended pollen seasons. The average temperature rises also increase risks of food- and water-borne diseases, and the transmission of arboviruses by mosquitoes. Loss of property and disruption to livelihoods and community cohesion due to extreme events also affect mental health and well-being. The health system is at the front line of these events and is burdened by both short- and long-term impacts of climate change. As such, our search strategy was guided by a systematic synthesis of reviews in the field of climate change [27,28], including a diverse range of climatic or environmental exposures (i.e., meteorological conditions, extreme events, ecosystem change, and air pollution), health outcomes (i.e., direct and indirect), and health services (i.e., emergency services, and health services needs and demands). We focused on Australian studies that considered the regional aspects of climate exposure in Northern NSW.

The literature search was conducted using the following electronic databases: MEDLINE (accessed on 2 September 2022), CINAHL (accessed on 4 September 2022), Scopus (accessed on 6 September 2022), Web of Science (accessed on 6 September 2022), and Informit (accessed on 7 September 2022). Table 1 shows the MeSH (Medical Subject Heading) terms and/or keywords used for the search strategy.

### 2.3. Eligibility Criteria

To be included, articles must have explicitly described the effects of climatic and environmental exposures on at least one specific direct or indirect human health outcome. We also included articles that evaluated the effects of climate change on health services.

We selected full-text peer-reviewed articles written in English, limited to records published in the past 10 years (January 2012–August 2022). Only articles that examined the effects of climate on health in Northern NSW were included; however, we initially considered articles from Australia and NSW more broadly as they may also describe aspects of climate change on health for our region of interest. We included a mix of evidence types, including systematic reviews, narrative reviews and original research.

### 2.4. Screening

The citations identified from the search strategy were imported into the reference manager Endnote for deduplication and subsequently into an online literature review tool, Covidence [29], for screening and data extraction. We applied a two-step screening process and included articles based on the eligibility criteria. The first screening was a single-reviewer screen of the title and abstract of each article by two reviewers. The second screening was a full-text screen completed independently by two reviewers. Any discrepancies between reviewers were discussed and resolved by the lead researcher.

### 2.5. Data Categorisation and Extraction

During the second screening stage, we categorised selected articles according to the following characteristics: type of study, geographical location, study population, climate exposure, health outcomes, or health services outcomes. The final selected articles were extracted and summarised for their main results, findings and study limitations.

### 2.6. Quality Assessment

To assess the risk of bias, we applied the National Health and Medical Research Council (NHMRC) levels of evidence [30] for all selected quantitative studies. For qualitative studies, we applied the hierarchy of evidence proposed by Daly et al., 2017 [31].

## 3. Results

### 3.1. Articles Selected

**Figure 3:**
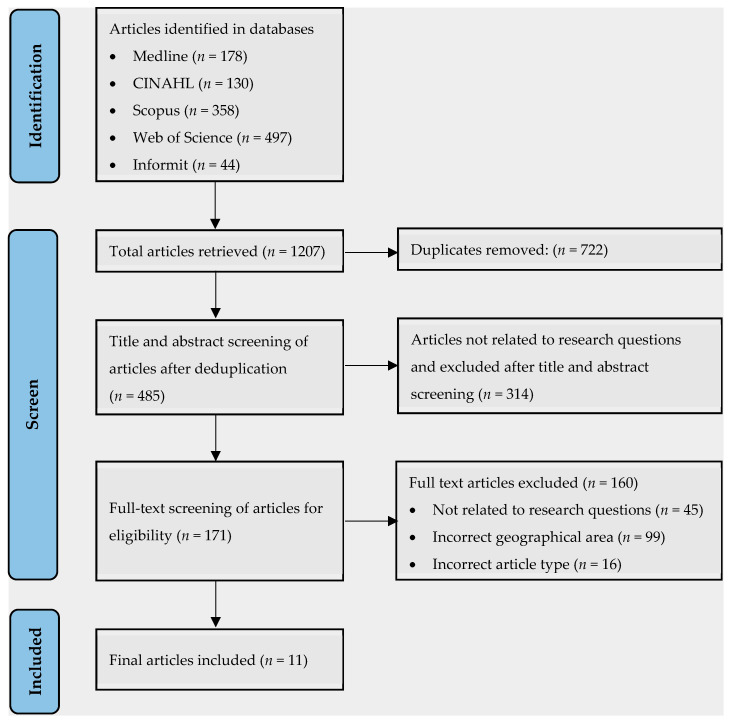
PRISMA flow chart showing the number of articles included or excluded in the rapid literature review stages.

The PRISMA flow chart Figure 3 shows the number of articles included and excluded in each stage of the review. From a total of 1207 peer-reviewed articles identified, we retained 11 articles for final inclusion. During the selection process, we excluded duplicated articles (*n* = 722) and those that were not relevant to the research questions by title and abstract screening (*n* = 314). During the full-text screening, articles were further excluded if they were not related to the research questions (*n* = 45), if the studies were not conducted in the relevant geographical location (*n* = 99), and if the articles were not reviews or original research articles (*n* = 16).

### 3.2. Study Characteristics

Table 2 summarises the characteristics of the selected articles (*n* = 11) that were published between 2012 and 2022. The majority of the studies were observational (9/11), including qualitative studies that analysed individuals’ experience of climate impacts (2/11), a descriptive study that described the change in mosquito abundance and human disease following environmental changes (1/11), and time-series, longitudinal, or cross-sectional studies that investigated associations between climate exposure and health outcomes (6/11). Only one study attributed health changes to long-term climate change and used scenario-based modelling to estimate the future health risk of climate exposure (1/11). No systematic reviews were retrieved, likely due to the small, regional focus of our research questions.

Regarding the geographical focus of the included studies, less than half of the studies specifically focused on the Northern NSW region (3/11). However, a further four studies examined climate exposures more broadly in NSW with consideration given to regions including Northern NSW (8/11).

The leading climate exposures investigated for Northern NSW were drought (5/11) and flood (3/11). One study investigated the effects of heat waves and cold waves, one study investigated bushfire and one study investigated meteorological parameters including rainfall and tidal waves on mosquito abundance.

In terms of health outcomes, most of the studies investigated mental health outcomes as a direct or indirect impact of flood or drought (8/11). One study targeted mortality as a heat- or cold-related health outcome, and one study tracked vector-borne disease notifications for Ross River virus (RRV) and Bamah Forest virus (BFV). Health system demand outcomes were reported in a small number of studies relating to general ambulance callouts and emergency department (ED) presentations, and cardiovascular- and respiratory-related ED visits (3/11).

### 3.3. Research Gaps

**Figure 4:**
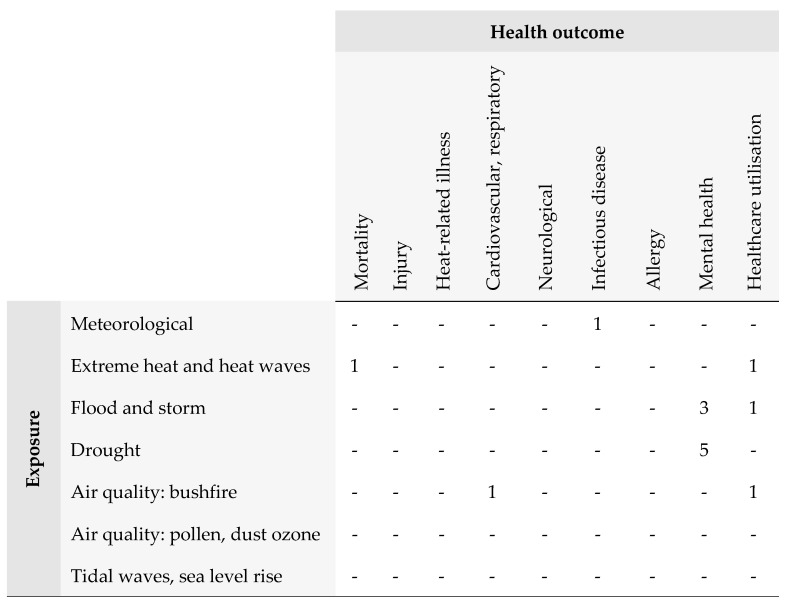
Number of studies found according to the main known climate exposure and health outcome categories.

Figure 4 highlights the evidence gaps, showing the number of studies found by the search strategy according to the main known categories of climate exposure and health outcomes [27,28]. For Northern NSW, no studies explored environmental exposures, such as pollen, dust, or tropospheric ozone, nor tidal waves or sea-level rise. For health outcomes, there were no studies that accounted for direct health impacts such as heat-related illness or accidental or occupational-related injuries. Indirect health impacts, such as water- and food-borne diseases, and other types of chronic diseases, such as neurological conditions and allergies, were missing. Mental health, although the most commonly explored climate-related health outcome, was not assessed in the context of bushfires. There was also a lack of studies on the impact of climate change on general mortality and healthcare utilisation due to other exposure types than bushfires and heat waves.

### 3.4. Summary of Findings

Overall, the selected studies observed adverse effects of a number of climate variables or extreme events with a wide variety of health outcomes. The majority of the studies analysed the association between recent variations of a climate variable (e.g., drought conditions between 2007–2013 [32,38]) or an occurrence of an extreme event (e.g., flood event in 2017 [35,36,37], bushfire event in 2019–2020 [41], and heavy rainfall in 2020 [34]) with health outcomes, such as mortality, mental health, and vector-borne diseases. Only a couple of studies used long-term datasets for evidence of change in rates of health outcomes that are associated with decadal changes of the climate parameter (e.g., heat/cold wave occurrence between 2005–2015 [43], drought conditions between 1970–2007 [39]); and only one study predicted the future rates of a health risk (i.e., suicidal risk 2000–2099 [42]) under future climate change scenarios. Table 3 summarises the main findings of included studies according to health outcomes. A further detailed summary of the findings is provided in Appendix A, Table A1.

#### 3.4.1. Mental Health

The most studied health outcome in relation to environmental and climate change in Northern NSW was mental health. The bulk of these mental health studies related to a cross-sectional survey of Northern NSW residents conducted six months after the major flooding in 2017. These studies showed that participants whose homes were inundated by the flood and/or displaced for more than six months after the flood had significantly greater odds of experiencing symptoms of post-traumatic stress disorder (PTSD), anxiety, and depression compared to those who were unexposed [37]. Participants whose homes were not inundated but who had disrupted access to social or health care also showed significantly greater odds of experiencing distress, PTSD, or anxiety [36]. Respondents from marginalised communities, including Aboriginal and Torres Strait Islander peoples, people who were in receipt of income support, and people with disability and their careers were found to be most at risk of having their homes inundated or being displaced, compounding existing social disadvantage and psychological morbidity [35,37].

The mental health burden of the drought was also reported as an important concern for Northern NSW. Regional and rural areas often experience prolonged periods of dryness that can contribute to the financial and psychological stress of farmers and communities. A longitudinal cohort study of non-metropolitan farmers who experienced the Millennium Drought of 1997–2010 found that being male, under 35 years of age, living and working on farms, financially insecure, and living in outer regional, remote or very remote NSW were at risk factors for general psychological distress [38]. Two qualitative studies capturing the perspectives and experiences of non-metropolitan farmers and residents found that floods, droughts and climate change contributed to emotional stress and anxiety [32,33]. Their concerns for the environment included water insecurity and the recurrent nature of disasters. Their emotional well-being would likely be impacted by financial loss; loss of material possessions and status; and loss of generational history and community structure. Communities also feared further decline in social and health infrastructure, which were already in limited supply due to the remoteness and small population sizes of outer regional and rural communities [32,33].

The mental health burden of the drought was also demonstrated in quantitative models. A time series Poisson generalised additive model of 11 rural and urban regions in NSW showed that during the historical period of 1970–2007, there was a substantial increase in suicide risk by 15% for rural males aged 30–49 years per annum for every increase in the interquartile range of the Hutchinson Drought Severity Index (HDSI)—an index that indicates rising duration and intensity of drought [39]. By applying the exposure–response function for drought and suicide established from the long-term data between 1970–2007, the attributable number (AN) of excess suicides per annum in drought was projected for the period 2000–2099 under modelled climate change scenarios [42]. Under the driest scenario in the projected period, an 84% increase in the AN of excess drought-related suicides for rural males aged 30–49 was estimated compared to the historical period. In contrast, there was no association between drought and suicide in urban populations [39,42].

#### 3.4.2. Vector Borne Disease

One ecological study observed the sequence of selected environmental signals, mosquito detections and human notifications of the Ross River virus (RRV) and Bamah Forest virus (BFV) in northeast NSW [34]. Following two extremely dry years and after an intense rainfall event and high tides in February 2020, there was a substantial increase in mosquito counts (after 2 weeks), followed by human notifications of RRV and BFV in northeast NSW (after 8 and 9 weeks, respectively) [34]. While the study did not model the quantitative relationship between environmental, entomological, and epidemiological events, the authors speculated that future climate change scenarios might result in more frequent and longer droughts followed by extreme rainfall, extending the timeframe in which mosquitoes can breed and increase the risk of human arboviral transmission in NSW [34].

#### 3.4.3. Mortality

A time-series analysis explored the health impacts of heat and cold waves on mortality in NSW and across three regions of remoteness: major cities, inner-regional, and outer-regional, remote and very remote areas [40]. The incidence rate ratio (IRR) of mortality was elevated in all of NSW after experiencing intense and very intense heat waves defined by the excess heat factor (EHF). Low temperatures were also associated with mortality. Intense cold waves, defined by the excess cold factor (ECF), were found to elevate the IRR of mortality in outer regional and remote areas [40].

#### 3.4.4. Health Services Utilisation

The associations between extreme events, including heat waves, cold waves, bushfires and floods with health service utilisation were explored in three studies. A time series analysis found a dose-response relationship between the severity of heat waves and ambulance callouts [40]. Increases in intensity levels of heat waves (indicated by EHF) were associated with increases in the IRR of ambulance callouts in NSW’s major cities, inner-regional areas, and outer-regional and remote areas [40]. The IRR of emergency department (ED) presentations was also elevated in each level of remoteness after intense heat waves. Cold waves were not associated with ambulance callouts, but the IRR of ED presentations were elevated in inner regional and outer regional and remote areas following intense and very intense cold waves [40].

Bushfire events were associated with increased use of healthcare services. A time-series analysis of the healthcare utilisation of the unprecedented summer bushfires of 2019–2020 in NSW found elevated relative risk (RR) of excess respiratory and cardiovascular- related ED visits for NSW [41]. For respiratory diseases, significantly elevated levels of ED visits were found in regions that experienced high fire intensity or were classified as low socioeconomic status (SES) [41]. However, this trend was not found for cardiovascular-related ED visits [41].

Health services utilisation can also be made inaccessible by extreme events. Cross-sectional analysis showed that the Northern NSW flood event in 2019 disrupted timely access to health and social services, particularly for people living with a disability [35]. Qualitative evidence from the study found that carers, as part of the support network for people living with a disability, had to move away from flood-impacted areas, thereby disrupting access to care [35]. Recovery support was perceived to be difficult to access, particularly for people living with a disability, from not being able to navigate the paperwork for accessing financial support to losing support from carers who were also impacted by the floods [35].

For the majority of the selected studies, the attribution of climate change on health was described speculatively without quantitative modelling. The predicted increase in frequency and intensity of extreme events was expected to exacerbate the climate-related health impact under investigation. Only one study used the relationship between climate exposure and health outcome underpinned by decadal long-term datasets, to quantify the magnitude of the health risk under different climate scenarios in the future.

### 3.5. Quality Assessment

For quantitative studies (9/11), their risk of bias is summarised in Appendix A, Table A2. All nine studies used Level III observational designs with moderate to high-levels of biases. These levels of biases call into question the studies’ internal validity and raise the need for more robust climate-related exposure and health outcome measurements and the estimation of their association.

For the qualitative studies (2/11), both were categorised as Level III descriptive studies, as summarised in Appendix A, Table A3. They described participant views or experiences by recording a range of illustrative quotes grouped under different thematic areas.

All the studies included in the review included a population from our study area of interest. These results are generalisable and applicable to other populations in similar climate settings in NSW. Moreover, the findings are applicable to other regional and rural settings in similar climatic zones with comparable topography in Australia.

## 4. Discussion

In this rapid review, we summarised the existing literature on the impacts of climate change on health and health services in Northern NSW. We categorised the evidence according to the climate exposure or health outcome topics and identified evidence gaps that may inform future research directions. Our search strategy retrieved a relatively small number of studies (*n* = 11), however, they do encompass a wide range of climate-related health topics considered important for Australia including heat waves, drought, bushfire smoke, heavy rainfall, and floods. This highlights the sensitivity of the Northern NSW region to the impacts of climate change.

### 4.1. Main Findings

Our review found that flooding is the most notable climate exposure studied for associated mental health impacts in the region. Indeed, Northern NSW experienced a cluster of major flood events in 2017 and 2022 [11]. During the 2022 flood event, the region received between 2.5–5 times the average rainfall in February compared to the 1961–1990 period [44]. More than 10,000 homes were damaged or destroyed, and 7000 people were displaced or in need of emergency accommodation [45]. While this review did not capture studies that explore the mental health impacts of the more recent 2022 floods, adverse mental health impacts were associated with the experience of the 2017 floods, particularly for marginalised communities. It is plausible that the clustered flood events can compound the mental health burden on affected individuals in the region. Future studies may investigate the compounding mental health impacts of clustered flood events.

Certain subpopulations in Northern NSW are more vulnerable to the mental health impacts of extreme events. For example, socioeconomically disadvantaged people and people with disability have lesser means to protect themselves, recover or access health services during or in the aftermath of flood events. The same applies to men from regional farming communities who are less financially able to recover from the loss of livelihood due to extended drought. There is evidence that individuals and communities traumatised by climate stress may further experience loss of identity and deteriorating social cohesion, particularly if they relocate to areas ill-equipped to support them [46]. Northern NSW also has a higher proportion of Aboriginal people compared to NSW overall. For Aboriginal people, connection to ‘Country’ is central to cultural well-being and witnessing environmental deterioration can be particularly harmful to their mental health and well-being [47,48,49].

Future research that provides a greater understanding of the risk profiles of vulnerable populations in the region will help LHDs provide more inclusive climate disaster planning [9,35]. This will enable more targeted actions when co-designing solutions with communities to prepare, respond or recover from extreme events. LHDs should work alongside local traditional owners who have cared for the country over generations, to listen and understand experiences of repeated disasters on their well-being, and design strategies for responsive and culturally sensitive healthcare. It is also important to have coordinated action across health levels for a holistic approach to mental health delivery for all populations, such as longer-term GP and psychological services support to affected individuals.

Our review identified studies that explored mortality, and cardiovascular- and respiratory-related health outcomes due to heat waves or bushfire-related smoke for regional NSW. Heat waves were associated with increase in mortality in Australia [50,51]; however, health impacts vary across communities and regions depending on the scale of the exposure and the range of health, social and built environment factors that modulate individual vulnerability [52]. For bushfire-related smoke, our review found that respiratory- and cardiovascular-related ED visits were elevated during bushfire periods across NSW, which is consistent with findings based on major Australian cities [53,54]. There was also evidence to show that respiratory-related ED visits were elevated in regions of high fire intensities and low SES. Socioeconomically disadvantaged people may have lesser means to afford protective measures or to move away from areas affected by extreme events. Future research that provides a greater understanding on the risk profiles of the region’s vulnerable populations to heat waves or bushfire smoke; and the patterns of related mortality or disease presentations at the hospital networks will support LHDs to better prepared for the expected increase in healthcare utilisation. Providing LHDs with locally relevant and timely air quality information during bushfire events will enable targeted, preventative public health messaging for vulnerable populations [55].

Finally, our review found one study that discussed the environmental and entomological events that brought about the most common mosquito-borne diseases in north-eastern NSW-RRV and BFV. In Australia, climate change is expected to favour temperature rise and tidal conditions that can increase the incidence and geographic distribution of arbovirus diseases [26]. However, these associations remained difficult to predict and each geographic region may have specific environmental or human behavioural factors that contribute to the level of risk [56]. The LHD can benefit from research that improves the sensitivity of arbovirus surveillance and mosquito monitoring system in the local area so that early detection of arboviruses can inform appropriate vector control or public health messaging strategies.

### 4.2. Research Gaps

The association between climate change and health is broad and complex. Our review focused on a diverse range of climatic and environmental exposures and health outcomes based on known impact pathways identified in the global climate change and health literature. For the Northern NSW region, we found that some exposure-health outcome associations were rarely explored. The LHD may further address these research gaps to gain a greater understanding of the climate-related health risks in their jurisdiction.

Our review did not identify studies that explored the direct health impact of extreme events, including heat waves, floods, and bushfires in Northern NSW. Excessively hot weather can elevate the risk of heat-related illnesses, such as heat stroke, heat exhaustion and occupational heat stress in outdoor settings, while floods and bushfires can directly cause accidental injury and death [57,58]. Furthermore, even though Northern NSW is a coastal region, our review did not identify studies investigating the health impact of tidal waves, storm surges or sea-level rise.

There is also a lack of studies that explore the wider spectrum of health consequences of climate change. Extreme events, such as bushfires, can also pose adverse long-term mental health impacts to affected communities in ways similar to flooding events. A study of the devastating 2009 Black Saturday bushfires in Victoria, Australia, found that people who lost properties reported PTSD and depression 3–4 years following the event [59]. For Northern NSW, a hotspot region of a range of environmental hazards [60], the compounding impacts of multiple extreme events occurring in succession, such as bushfires, droughts, and floods, should be considered.

As the average temperature increases with climate change, the risk of exposure to food-borne enteric pathogens, such as Salmonella, may increase in summer [61]. Heavy rainfall, flood and drought can adversely affect the quality of drinking water, introducing pathogens, such as Cryptosporidium or *E. coli* [62,63]. Sea-level rise and storm surges can also increase the risk of salt-water intrusion in the drinking water supply. Climate change may worsen air quality and respiratory health in other ways. More frequent heavy rain and flood events can increase moisture levels and promote fungal exposure inside homes or liberate toxic substances, such as asbestos, lead and arsenic [64]. Pollen seasons may become more prolonged under a warming climate, which can exacerbate seasonal allergic rhinitis and asthma [64]. Other potential climate-related health outcomes, such as neurological diseases or emerging infectious diseases (e.g., zoonotic disease including Q fever, vector-borne disease including Japanese encephalitis), may also increase under climate change.

Finally, the impact of climate change on the health system or healthcare utilisation is less well studied for the Northern NSW region. While there were indications that extreme events can elevate emergency healthcare utilisation, there were no studies that assessed their physical impact on hospital facilities, critical health infrastructure and healthcare supply chains.

### 4.3. Research Directions

Our search strategy aimed to capture research studies that assessed the observed impact of climate on health and the predicted future health impacts. We found that most of the studies were epidemiological studies that explored the relationship between recent past climate events and health outcomes. This perhaps illustrates the difficulty in undertaking statistical modelling that predicts future health burdens or surges in healthcare utilisation under a changing climate. The challenges include the unavailability of long-term observational data; complexities in accounting for a wide range of non-climatic factors that can also influence vulnerability to climate change; and uncertainties inherent to predicting climatic, demographic and socio-economic states [65,66]. Despite the complexities, climate change and health modelling are important research priorities as they can inform decision-making, risk communication, early warning systems and the co-planning of adaptation measures with at-risk population groups.

Section 4.1 and Section 4.2 of the discussion outlined some knowledge gaps that the NNSW LHD can address to gain a greater understanding of the climate-related health risks in their jurisdiction. Aside from understanding the scale and nature of climate-related health threats, there are also other research directions LHDs may consider. The WHO provided a frame of reference for research priorities that can be considered in the field of climate change and health [67]. Of relevance to LHDs are research that investigates the cost-effectiveness of health protection strategies relating to climate change, or the financial cost and necessary resources required to protect health. There is also scope for in-depth, qualitative research that aims to understand how the public or the health workforce perceives climate-related health risks or how they can be engaged to co-design climate mitigation or adaptation solutions.

Finally, whole-of-system research is necessary to detail the roles of emergency services, primary care, routine care, and preventative health services under a changing climate [9,68,69,70,71,72,73,74]. This entails identifying contributions each can make to patient care, public health, psychological support, and other specialised tasks across all phases of disasters (planning, preparedness, response, and recovery). Whole-of-system approach will require support from the state and national governments so that climate-resilient healthcare can be implemented at the local level. The whole-of-system approach also encompasses cross-sectoral responses. For example, under a warming climate, people living in poor-quality housing are subjected to a greater risk of heat-related illness, infectious disease and climate-related chronic health burdens [75,76,77]. Working with the housing sector can bring about locally relevant guidelines and policies that better support health in the long term.

### 4.4. Limitations

This rapid review has a number of limitations. First, the study region under investigation is relatively small, which substantially limits the number of studies found. This also has consequences on needing to exclude systematic reviews that aggregate evidence from a greater geographical scale which would otherwise provide a more robust understanding of the specific exposure-health outcome association. However, our rapid review synthesises individual original studies that provided relevant information for the region under investigation. Second, our search query was built to focus on climate change and human health that may not fully capture all the direct and indirect impacts that climate change can have on health, particularly regarding the literature that investigates indirect health impacts that are not necessarily described within the context of climate change, such as impacts related to climate-sensitive air pollutants, poor housing condition, food and water insecurity, and displacement.

## 5. Conclusions

Unmitigated climate change will exacerbate a wide range of climate-related human health risks and disproportionately impact people who are already socio-economically marginalised. At the same time, climate change will undermine public health and healthcare systems, particularly in resource-poor regional areas. The need for LHDs to prepare for the effects of climate change and implement whole-of-system responses at the local level are emerging. Our rapid review found a relatively small number of studies that explored the impacts of climate change on health and health services. Most studies explored the mental health outcomes of floods and droughts, followed by increased healthcare utilisation due to respiratory, cardiovascular, or mortality outcomes of bushfires or heat waves; and arbovirus outbreaks during wet summer seasons. Significant research gaps include climate-related health outcomes, such as injury, heat-related illness, water- and food-borne diseases, and other chronic health conditions; health system impacts; and population exposure to bushfire smoke and dust and rising sea levels. There is also a lack of predictive modelling of future health risks and healthcare utilisation for the region.

Future studies should address the identified research gaps. Moreover, the LHD will benefit from understanding the compounding impacts of multiple extreme events on health and the health system; local data needs required to inform predictive models of health risks; the risk profiles of populations vulnerable to the different climate-related exposures extreme events; and whole-of-system response required to mitigate the impact. In order to engender an actionable research agenda, the LHD will need to prioritise research in alignment with their institutional capacities and strategic directions. The research directions our rapid review provide may inform future adaptation and mitigation policies and strategies relevant to the region.

## Figures and Tables

**Table 1:** Search terms used to identify research articles that report on the impact of climate change on health and health services in Northern NSW (example of the MEDLINE search).

Key Concept	Keywords
Climate change	(exp climate change/OR (climate change * or global warm * or sea level ris *).mp.) AND (cyclonic storms/OR (cyclon * or storm *).mp. OR droughts/OR drought *.mp. OR floods/OR flood *.mp. OR lightning/OR lightning.mp. OR rain/OR (rain * or extreme rain *).mp. OR tidal waves/OR tid * wave *.mp. OR exp natural disasters/OR (natural disaster * or landslide * or wildfire * or bushfire * or landscape fire or fire storm * or tornado *).mp. OR air pollutants/OR (air pollutant * or particulate matter or dust or pollen or ozone).mp. OR air pollution/OR air pollution.mp. OR exp temperature/OR (hot temperature or heat wave or extreme heat).mp. OR extreme weather/OR extreme weather.mp. OR humidity/OR humid *.mp.) AND
Human health and	exp health/OR health.mp. OR health equity/OR health equity.mp. OR disease/OR (disease * or cardiovascular or respiratory).mp. OR exp mortality/OR (mortal * or death or premature death or fatal *).mp. OR mental health/OR (mental health or well?being).mp. OR anxiety/OR anxiety.mp. OR depression/OR depress *.mp. OR exp heat stress disorders/OR (heat stress or heat stroke * or heat?related illness * or heat exhaustion).mp. OR exp vector borne diseases/OR (vector?borne disease * or mosquito?borne disease * or arbovirus infection *).mp. OR
Health services	exp “health care facilities, manpower, and services”/OR (health service * or community health * or (emergency adj2 service *) or mental health service * or rural health or regional health or urban health or aboriginal health or general practice or public health).mp. OR (workforce * or health infrastructure or supply chain * or transport or health plan *).mp. OR “health services needs and demand”/OR (health * adj3 demand *).mp. OR health servic *.mp. OR Ambulatory care/OR (ambulatory care or ambulance *).mp. OR disasters/or disaster planning/or emergencies/or emergency shelter/OR (disaster * or disaster plan * or emergenc * or emergency shelter *).mp. AND
Australia and Northern NSW	exp Australia/OR (Australia * or New South Wales or Northern Territory or Western Australia or South Australia or Victoria or Tasmania or Queensland or Australian Capital Territory or Torres Strait Island *).mp. OR New South Wales/OR (New South Wales or NSW or local government or “Northern New South Wales Local Health District” or “Northern New South Wales” or Northern New South Wales or east coast or north coast or Northern Rivers or Lismore).mp.

exp/retrieves references related to the subject heading and relevant sub-topics; .mp. retrieves references when the word appears in specific fields including title, abstract, subject heading, author keywords and more; * retrieves unlimited suffix variations; ? replaces zero or one character; adj searches for two words or phrases that appear within a set number of words of each other.

**Table 2:** Characteristics of selected studies.

Characteristics		Number (*n* = 11)	References
Type of study	Qualitative	2	[32,33]
	Descriptive	1	[34]
	Cross-sectional	3	[35,36,37]
	Longitudinal cohort	1	[38]
	Time-series	3	[39,40,41]
	Detection and attribution model	1	[42]
Location	State level with a focus on regional areas	8	[32,33,34,38,39,40,41,42]
	Northern NSW	3	[35,36,37]
Climate exposure	Meteorological	1	[34]
	Extreme heat and heat wave	1	[40]
	Flood	3	[35,36,37]
	Drought	5	[32,33,38,39,42]
	Bushfire and air quality	1	[41]
Health outcome	All-cause mortality	1	[40]
	Infectious diseases (vector-, food-, water-borne)	1	[34]
	Respiratory, cardiovascular	1	[41]
	Mental health	8	[32,33,35,36,37,38,39,42]
Health system	Health services	3	[35,40,41]

**Table 3:** Summary of findings of included studies (*n* = 11).

Health Outcome	*n*	Climate Exposure	Location	Summary of Findings
Mental health	8	Flood and storm	Northern NSW	Cross-sectional analyses found that the 2017 flood event in Northern NSW was associated with probable post traumatic stress disorder (PTSD), anxiety, and depression, particularly for people whose homes, businesses, and farms were inundated, displaced [37], and particularly for marginalised communities (e.g., people with a disability) [35,36]. The disruption of access to healthcare and social services due to floods was also associated with probable PTSD [36].
		Drought	NSW with a regional focus	A time series analysis found an association between drought and increased risk of suicide in the period of 1970–2007, particularly for male farmers from rural regions of NSW [39]. Modelling of future climate scenarios found that an increase in the duration and intensity of droughts will increase suicide rates among males in rural NSW between 2000–2099 [42]. A longitudinal study found that younger farmers that experienced the Millennium Drought of 1997–2010 in regional NSW were more likely to report drought-related stress [38]. Qualitative studies found that concerns for the environmental, financial, health and social impacts of climate change may impact the mental health and well-being of rural communities [32,33]
Health service utilisation	3	Flood	Northern NSW	A cross-sectional analysis found that access to healthcare and social services were disrupted during and after the 2017 flood event for affected communities in the Northern Rivers, particularly for people with disabilities and for carers [35].
		Heat waves	NSW with a regional focus	A time series study found that heat waves were associated with greater ambulance callouts and emergency department (ED) presentations in the period of 2005–2015 across urban, regional and remote areas of NSW [40].
		Bushfire	NSW with a regional focus	A time-series study found that the 2019–2020 bushfire elevated the use of ED visits due to cardiovascular and respiratory conditions. Respiratory related-ED visits were elevated in regions of lower socioeconomic status and higher fire densities [41].
Vector-borne disease	1	Rainfall	Northern NSW	A descriptive study found that Ross River virus (RRV) and Bamah Forest virus (BFV) human disease notifications increased in northeast NSW following a significant rainfall event, high tides, and substantial increase in mosquito abundance in the summer of February 2020 [34].
Mortality	1	Heat waves	NSW with a regional focus	A time series analysis found an association between heat waves and mortality across urban, regional, and remote areas of NSW in the period between 2005–2015 [40].
Cardiovascular and respiratory	1	Bushfire	NSW with a regional focus	A time-series study found that the 2019–2020 bushfire found increased cardiovascular and respiratory-related ED visits [41].

## Data Availability

All relevant data within the manuscript are available from the corresponding author upon reasonable request.

## Abbreviations and Acronyms

BFV: Bamah Forest virus; ED: emergency department; EHF: excess heat factor; ECF: excess cold factor; FFDI: Forest Fire Danger Index; IRR: incidence rate ratio; LHD: Local Health District; MeSH: Medical Subject Heading; NARCliM: NSW and ACT Regional Climate Modelling project; NSW: New South Wales; PRISMA: Preferred Reporting Items for Systematic Review and Meta-Analysis; PTSD: post-traumatic stress disorder; RR: relative risk; RRV: Ross River virus; SES: socioeconomic status.

## Appendix A

**Table A1:** Detailed summary of findings (*n* = 11).

First Author	Year	Title	Location	Population/ Participants	Study Type/Period	Exposure/ Outcome	Key Findings	Limitations
Austin et al. [38]	2018	Drought-related stress among farmers: findings from the Australian Rural Mental Health Study.	NSW non-metropolitan regions (inner regional, outer regional, remote, very remote regions).	Adults living and/or working on a farm (*n* = 664) aged >18 from the Australian Rural Mental Health Study (ARMHS) Predominately live on farms in inner and outer regional areas.	Longitudinal cohort ARMHS 2007–2013 Generalised Estimating equations model.	Exposure: drought (rainfall deficiency) Outcome: mental health (generalised psychological distress K10 scale, Personal Drought-related Distress (PDS) and Community Drought-related Distress (CDS) Likert scale).	Significant: Compared to those who live on a farm only, those who live and work on a farm were likely to experience PDS (IRR = 1.50; 95% CI: 1.32–1.72). Significant: Compared to female farmers, male farmers were likely to experience elevated general psychological distress. Significant: Compared to older farmers >35, farmers aged 18–35 were likely to experience elevated PDS and CDS. Significant: Compared with farmers from inner regional areas, farmers from outer regional and remote and very remote areas were likely to experience elevated PDS (outer regional IRR = 1.88, 95% CI: 1.59–2.23; remote IRR = 2.02, 95% CI: 1.65–2.48); very remote IRR = 2.55, 95% CI: 2.55, 95% CI = 1.97–3.30) and CDS (outer regional IRR = 2.05, 95% CI: 1.76–2.38; remote IRR = 2.17, 95% CI: 1.83–2.58; very remote IRR = 2.80, 95% CI: 2.26–3.47). Significant: Compared to farmers with comfortable financial status, farmers with lower financial security were likely to experience elevated general psychological distress (IRR = 2.99, 95% CI = 1.52–5.89) and PDS (IRR = 1.72, 95% CI = 1.38–2.14).	Application of the meteorological definition of drought may miss out on mental health effects captured by other measures of drought type. Self-reporting of health outcomes can introduce bias and misclassification.
Austin et al. [32]	2020	Concerns about climate change among rural residents in Australia.	NSW non-metropolitan regions (inner regional, outer regional, remote, and very remote regions).	Adults from non-metropolitan NSW (*n* = 823) Age > 18 ARMHS Participants.	Open-ended questionnaire. Word frequency test, thematic analysis 2007–2013.	Exposure: drought, climate change Outcome: perspectives and concerns.	Qualitative: Many respondents believed climate change was natural and not caused by human actions. Key concerns were the financial, environmental (particularly water insecurity), and health and social impacts. Both governments’ inaction to mitigate climate change and the cost of such actions were of concern.	Analysis of free text based on an open-ended questionnaire may not capture in-depth opinions.
Bailie et al. [35]	2022	Exposure to risk and experiences of river flooding for people with disability and carers in rural Australia: a cross-sectional survey.	Northern NSW (6 Local Government Areas: Ballina Shire, Tweed Shire, Richmond Valley, Kyogle, Byron Shire, and Lismore City).	Adults (*n* = 2252) Age > 16 Predominately female, aged >45; People with disability (*n* = 165); Carers (*n* = 91).	Snowball, cross-sectional survey, 6 months post-flood event in 2017.	Exposure: flood event Outcome: mental health.	Significant: Compared to other respondents, the odds of having homes flooded were elevated for people with disability (OR = 2.41, 95% CI: 1.71–3.39) or carers (OR = 1.76, 95% CI: 1.10–2.84). The odds of having to evacuate and be displaced for more than 6 months were elevated for people with disability (OR = 3.78, 95% CI: 2.18–6.55). The odds of not getting timely help or access to health and social services were elevated for people with disability (OR = 3.98%, 95% CI = 2.82–5.60). Significant: Compared to other respondents, the odds of experiencing probable PTSD elevated for carers or people with disability (OR = 3.32, 95% CI: 2.22–4.96).	Limited generalisability. Difficult to establish causation. Self-reporting of mental health conditions can introduce bias and misclassification.
Hanigan et al. [39]	2012	Suicide and drought in New South Wales, Australia, 1970–2007.	NSW (11 rural or urban regions, including Northern NSW.	Age > 10 by males and females.	Time series 1970–2007 Poisson generalised additive model.	Exposure: drought (Hutchinson Drought Severity Index HDSI) Outcome: suicides.	Significant: Increase in relative risk of suicide by 15% (95% CI: 8–22%) in rural males aged 30–49 years per the first to third quartile increase in the HDSI. The predicted annual suicides in this subgroup was 4 (95% CI: 2.14–6.05) or 9% of the total number in the age group). Significant: Decrease in relative risk of suicide for rural females aged 30–49 (RR = −0.72, 95% CI: −0.31 to −0.01). No association between drought and suicide in urban populations.	Likely underestimation of historical suicide risk in farmers.
Hanigan et al. [42]	2022	Climate Change, Drought, and Rural Suicide in New South Wales, Australia: Future Impact Scenario Projections to 2099.	NSW (11 rural or urban regions, including Northern NSW.	Age > 10 by males and females.	Modelling Baseline: 1970–1999; Projection: 2000–2099.	Exposure: drought (HDSI) Outcome: suicides.	Significant: Compared to the attributable number (AN) of excess suicides per annum in drought for rural males aged 30–49 in the historical period, an 84% increase (95% CI: 2–159) was projected under the driest scenario RCP 4.5 (AN = 1.5, 95% CI: 0.84–2.13). Significant: Decrease in AN of excess suicides per annum in drought for rural females aged 30–49 between historical (AN = −0.29, 95% CI: −0.61 to −0.03) and projected (AN = −0.53, 95% CI: −1.14 to −0.05) period. Significant: Elevated gender differences in AN of excess suicides per annum in drought between rural males and females aged 30–49 in the projected period (AN = 2.04, 95% CI: 1.98–2.18). No gender difference in drought-related suicide rates in urban populations.	Limited assessment of regional patterns in the modelled climate data may introduce exposure misclassification bias. Did not consider the effect of temperature on suicide.
Hime et al. [34]	2022	Weather extremes associated with increased Ross River virus and Barmah Forest virus notifications in NSW: learnings for public health response.	NSW east coast/northeast NSW (4 major population centres on central, mid-north, and north coast of NSW).	NSW residents of northeast NSW.	Ecological Descriptive analysis February 2020.	Exposure: High rainfall, high tides, high mosquito counts Outcome: RRV/BFV detections and Human RRV/BFV notifications.	Descriptive: Following two extremely dry years and after a significant rainfall event and high tides in February 2020, there was a substantial increase in mosquito abundance occurred after 2 weeks, followed by RRV and BFV notifications in northeast NSW after 8 and 9 weeks, respectively. Mosquito bite avoidance messaging should be instigated within 2 weeks of high summer rainfall, especially after an extended dry period.	Environmental, entomological, and epidemiological events may not necessarily be linked. Underestimation of human infections by surveillance system due to asymptomatic infections.
Jagasothy et al. [40]	2017	Extreme climatic conditions and health service utilisation across rural and metropolitan New South Wales.	NSW (3 regions: major cities, inner regional and outer regional /remote/very remote areas).	NSW residents.	Time-series 2005–2015 Poisson regression model.	Exposure: Cold wave, heat wave Outcome: mortality, ambulance callouts, and emergency department (ED) presentations.	Significant: A positive dose–response relationship was observed between the severity of heat waves and ambulance callouts in each study region. Significant: Compared with a non-heat wave days, incidence rate ratio (IRR) of ambulance call-outs were elevated in NSW after low-intensity (IRR = 1.018, 95% CI: 1.015–1.022), intense (IRR = 1.047, 95% CI: 1.039–1.056) and very intense (IRR = 1.109, 95% CI: 1.077–1.142) heat waves. This was also the case for each study region. Significant: ED presentations were also elevated in NSW after low-intensity (IRR = 1.009, 95% CI: 1.004–1.014) and intense (IRR = 1.036, 95% CI: 1.024–1.048) heat waves. Significant: Mortality was elevated after intense (IRR = 1.024; 95% CI: 1.008–1.041) and very intense (IRR = 1.108; 95% CI: 1.045–1.174) heat waves for NSW. This is also the case for major cities. This maybe due to the urban heat island effect. Significant: ED presentations were elevated after low (IRR = 1.047; 95% CI: 1.042–1.052); intense (IRR = 1.093; 95% CI: 1.080–1.106) and very intense (IRR = 1.082; 95% CI: 1.050–1.116) cold waves in outer regional/remote/very remote areas.	
King et al. [36]	2020	Disruptions and mental health outcomes following Cyclone Debbie.	Northern NSW (6 Local Government Areas: Ballina Shire, Tweed Shire, Richmond Valley, Kyogle, Byron Shire, and Lismore City).	Adults (*n*= 2180) Age > 16 Predominately female, aged >45; Direct disruption (*n* = 1228); Indirect disruption (*n* = 605).	Snowball, cross-sectional survey, 6 months post-flood event in 2017.	Exposure: flood event Outcome: Mental health, loss of access to social/health care, food, and utilities.	Significant: Compared to non-disrupted respondents, odds of experiencing distress (OR = 3.31; 95% CI: 1.79–6.12), probable PTSD (OR = 13.48; 95% CI: 5.45–33.35), depression (OR = 4.26; 95% CI: 2.35–7.73), anxiety (OR = 3.64; 95% CI: 1.74–7.62), and suicidal ideation (OR = 2.86; 95% CI: 1.36–5.99) were elevated for directly disrupted respondents. Mental health outcomes of indirectly disrupted respondents were less severe, but the odds of experiencing probable PTSD (OR = 3.52, 95% CI: 1.36–9.15) were still elevated. Significant: Compared to non-disrupted respondents, odds of experiencing distress (OR = 1.86, 95% CI: 1.38–2.49), probable PTSD (OR = 1.93, 95% CI: 1.38–2.7), anxiety (OR = 2.67, 95% CI: 1.64–4.35) and suicidal ideation (OR = 1.74, 95% CI: 1.14–2.66) were elevated for those who lost access to social services/healthcare.	Limited generalisability. Difficult to establish causation. Self-reporting of mental health conditions can introduce bias and misclassification.
Matthews et al. [37]	2019	Differential mental health impact six months after extensive river flooding in rural Australia: A cross-sectional analysis through an equity lens.	Northern NSW (6 Local Government Areas: Ballina Shire, Tweed Shire, Richmond Valley, Kyogle, Byron Shire, and Lismore City).	Adults (*n*= 2180) Age > 16 Predominately female, aged 35–74.	Snowball, cross-sectional survey, 6 months post-flood event in 2017.	Exposure: flood event Outcome: mental health.	Significant: Compared to unexposed respondents, odds of experiencing distress (OR = 25.70, 99% CI: 9.20–71.81), probable PTSD(OR = 24.43, 99% CI: 7.05–84.69), anxiety (OR = 14.50, 99% CI: 5.15–40.85), and depression (OR = 8.38, 99% CI: 3.04–23.10) were elevated for respondents who were displaced after 6 months. This was also the case for those whose homes/businesses/farms were evacuated/flooded. Significant: Odds of experiencing probable anxiety (OR = 2.16, 99% CI: 1.08–4.33) and depression (OR = 2.09, 99% CI: 1.04–4.23) were elevated for Aboriginal and Torres Strait Islanders. This was also the case for income support recipients’ respondents (anxiety OR = 1.89, 99% CI: 1.26–2.85; depression OR = 1.84, 99% CI: 1.22–2.79).	Limited generalisability. Difficult to establish causation. Self-reporting of mental health conditions can introduce bias and misclassification.
Ng et al. [33]	2015	Climate adversity and resilience: the voice of rural Australia.	NSW (2 regional centres, 2 rural villages).	Adults who experienced drought and flood within 5 years Age > 18 (*n* = 46).	In-depth focus groups and interviews Purposive and convenience sampling.	Exposure: drought and flood Outcome: experience, health, and wellbeing.	Qualitative: Flood and drought contributed to emotional stress and anxiety due to fear about the reoccurrence of the disasters, financial loss; loss of possession; loss of status, family heritage, generational history, and rural community structure; loss of material procession; loss of physical infrastructure. Social connectedness can promote resilience in disaster-struck rural communities.	Limited generalisability of research findings to the broader rural population due to differences in climate, farming practices, community, and rural culture.
Wen et al. [41]	2022	Excess emergency department visits for cardiovascular and respiratory diseases during the 2019–2020 bushfire period in Australia: A two-stage interrupted time-series analysis.	NSW (28 Statistical Area 4 regions in NSW).	NSW residents.	Time-series Two-stage interrupted analysis 2019–2020 bushfire period.	Exposure: Bushfire event Outcome: respiratory and cardiovascular-related ED visits.	Significant: Compared to the same periods of 2017–2018 and 2018–2019, the relative risk (RR) of excess ED visits for respiratory (RR = 1.05, 95% CI: 1.02–1.09) and cardiovascular diseases (RR = 1.10, 95% CI: 1.07–1.13) were elevated during the 2019–2020 bushfires. Significant: The RR of excess respiratory -related ED visits were elevated in low SES regions compared to high SES regions (RR = 1.12, 95% CI: 1.06–1.18); and in regions of high fire intensity compared to low fire intensity. For cardiovascular-related ED visits, no significant difference was found when stratified by SES and fire intensity.	Outcome classification maybe introduced in diagnostic outcome data as they were not coded by clinicians.

**Table A2:** Quality assessment of quantitative studies using the NHMRC level of evidence tool.

Author, Year, Title	Level of Evidence	Consistency of Evidence	Clinical Impact	Risk of Bias	Generalisability and Applicability
Austin et al., 2018 [38] Drought-related stress among farmers: findings from the Australian Rural Mental Health Study.	Level III-2 *	Mostly consistent with other studies and inconsistency may be explained.	Very large	Moderate	Population/s studied in body of evidence are the same as the target population in question. Directly applicable to Australian healthcare context.
Bailie et al., 2022 [35] Exposure to risk and experiences of river flooding for people with disability and carers in rural Australia: a cross-sectional survey.	Level III-2 *	Mostly consistent with other studies and inconsistency may be explained.	Very large	High	Population/s studied in body of evidence are the same as the target population in question. Directly applicable to Australian healthcare context.
Hanigan et al., 2012 [39] Suicide and drought in New South Wales, Australia, 1970–2007.	Level III-2 *	Consistent with other studies.	Moderate	Moderate	Population/s studied in body of evidence are the same as the target population in question. Directly applicable to Australian healthcare context.
Hanigan et al., 2022 [42] Climate Change, drought and rural suicide in New South Wales, Australia: Future impact scenario projections to 2099.	Level III-2 *	Consistent with other studies The authors discussed in detail the similarity with other study findings and with the findings from their previous studies.	Moderate	Moderate	Population/s studied in body of evidence are the same as the target population in question. Directly applicable to Australian healthcare context.
Hime et al., 2022 [34] Weather extremes associated with increased Ross River virus and Barmah Forest virus notifications in NSW: learnings for public health response.	Level III-2 *	Consistent with other studies.	Very large	Moderate	Population/s studied in body of evidence are the same as the target population in question. Directly applicable to Australian healthcare context.
Jagasothy et al., 2017 [40] Extreme climatic conditions and health service utilisation across rural and metropolitan New South Wales.	Level III-2 **	Mostly consistent with other studies and inconsistency may be explained.	Very large	Low	Directly applicable to Australian healthcare context.
King et al., 2020 [36] Disruptions and mental-health outcomes following Cyclone Debbie.	Level III-3 **	Mostly consistent with other studies and inconsistency may be explained.	Very large	High	Population/s studied in body of evidence are the same as the target population in question. Directly applicable to Australian healthcare context.
Matthews et al., 2019 [37] Differential Mental Health Impact Six Months After Extensive River Flooding in Rural Australia: A Cross-Sectional Analysis Through an Equity Lens.	Level III-2 *	Mostly consistent with other studies and inconsistency may be explained.	Very large	Moderate	Population/s studied in body of evidence are the same as the target population in question. Directly applicable to Australian healthcare context.
Wen et al., 2022 [41] Excess emergency department visits for cardiovascular and respiratory diseases during the 2019–2020 bushfire period in Australia: A two-stage interrupted time-series analysis.	Level III-2 *	Mostly consistent with other studies and inconsistency may be explained.	Moderate	Low	Populations studied in body of evidence are the same as the target population in question. Directly applicable to Australian healthcare context.

* Level III-2: A comparative study with concurrent controls. For example, non-randomised experimental trials, cohort studies, case-control studies, interrupted time series studies with a control group. ** Level III-3: A comparative study without concurrent controls. For example, historical control study, two or more single-arm studies, interrupted. time series studies without a parallel control group.

**Table A3:** Quality assessment of qualitative studies using hierarchy of evidence for assessing qualitative health research tool.

Author, Year, Title	Level of Evidence	Risk of Bias	Evidence for Practice
Austin et al., 2020 [32] Concerns about climate change among rural residents in Australia.	Level 3 Descriptive studies	Do not report full range of responses.	Demonstrate that a phenomenon exists in a defined group. Identify practice issues for further consideration.
Ng et al., 2015 [33] Climate adversity and resilience: the voice of rural Australia.	Level 3 Descriptive studies	Do not report full range of responses.	Demonstrate that a phenomenon exists in a defined group. Identify practice issues for further consideration.
